# Early treatment with FCR versus watch and wait in patients with stage Binet A high-risk chronic lymphocytic leukemia (CLL): a randomized phase 3 trial

**DOI:** 10.1038/s41375-020-0747-7

**Published:** 2020-02-18

**Authors:** Carmen D. Herling, Florence Cymbalista, Carolin Groß-Ophoff-Müller, Jasmin Bahlo, Sandra Robrecht, Petra Langerbeins, Anna-Maria Fink, Othman Al-Sawaf, Raymonde Busch, Raphael Porcher, Bruno Cazin, Brigitte Dreyfus, Stefan Ibach, Stéphane Leprêtre, Kirsten Fischer, Florian Kaiser, Barbara Eichhorst, Clemens-Martin Wentner, Manuela A. Hoechstetter, Hartmut Döhner, Veronique Leblond, Michael Kneba, Remi Letestu, Sebastian Böttcher, Stephan Stilgenbauer, Michael Hallek, Vincent Levy

**Affiliations:** 1 Department I of Internal Medicine and Center of Integrated Oncology Aachen Bonn Cologne Duesseldorf, University of Cologne, Cologne, Germany; 2 Hopital Avicenne, Assistance Publique–Hopitaux de Paris (AP-HP), Service d’Hematologie Biologique, Bobigny, France; 3 Institute for Medical Statistics and Epidemiology, Technical University, Munich, Germany; 4 Center of Clinical Epidemiology, Hopital Hotel-Dieu, Paris, France; 5 Service de Maladies du Sang, CHU Claude Huriez, Lille Cedex, France; 6 Service d’Hematologie, CHU Poitiers, Poitiers, France; 7 WiSP Wissenschaftlicher Service Pharma GmbH, Langenfeld, Germany; 8 Inserm Unit U1245 and Department of Hematology, Cancer Centre Henri Becquerel and Normandie University Rouen, Rouen, France; 9 Day Clinic Hematology Oncology Palliative Care, Landshut, Germany; 10 Department of Hematology Oncology, Immunology, Palliative Medicine, Infectious Diseases and Tropical Medicine, Klinikum Schwabing, Munich, Germany; 11 Department of Internal Medicine III, University of Ulm, Ulm, Germany; 12 Department of Haematology, Hopital de la Pitie-Salpetriere, Paris, France; 13 Second Department of Medicine, University of Schleswig-Holstein, Kiel, Germany; 14 Cluster of Excellence on Cellular Stress Responses in Aging-associated Diseases (CECAD), Cologne, Germany; 15 Unite de Recherche Clinique, Hopital Avicenne, Bobigny, France; 16 Present Address: Department of Hematology, Oncology and Palliative Medicine, Center for Internal Medicine, University of Rostock, Rostock, Germany

**Keywords:** Randomized controlled trials, Chronic lymphocytic leukaemia

## Abstract

We report a randomized prospective phase 3 study (CLL7), designed to evaluate the efficacy of fludarabine, cyclophosphamide, and rituximab (FCR) in patients with an early-stage high-risk chronic lymphocytic leukemia (CLL). Eight hundred patients with untreated-stage Binet A disease were enrolled as intent-to-treat population and assessed for four prognostic markers: lymphocyte doubling time <12 months, serum thymidine kinase >10 U/L, unmutated IGHV genes, and unfavorable cytogenetics (del(11q)/del(17p)/trisomy 12). Two hundred and one patients with ≥2 risk features were classified as high-risk CLL and 1:1 randomized to receive either immediate therapy with 6xFCR (Hi-FCR, 100 patients), or to be observed according to standard of care (Hi-W&W, 101 patients). The overall response rate after early FCR was 92.7%. Common adverse events were hematological toxicities and infections (61.0%/41.5% of patients, respectively). After median observation time of 55.6 (0–99.2) months, event-free survival was significantly prolonged in Hi-FCR compared with Hi-W&W patients (median not reached vs. 18.5 months, *p* < 0.001). There was no significant overall survival benefit for high-risk patients receiving early FCR therapy (5-year OS 82.9% in Hi-FCR vs. 79.9% in Hi-W&W, *p* = 0.864). In conclusion, although FCR is efficient to induce remissions in the Binet A high-risk CLL, our data do not provide evidence that alters the current standard of care “watch and wait” for these patients.

## Introduction

Clinical observation without therapy—defined as “watch and wait” (W&W)—has been the gold standard for the management of early-stage chronic lymphocytic leukemia (CLL). This principle is based on the repeated failure of previous attempts to improve the clinical outcome of CLL patients by early therapeutic intervention [1–4]. Moreover, a reasonable subset of patients with CLL experience an indolent disease course with neither compromising morbidity nor an elevated risk of premature death caused by the leukemia. Such patients have a life expectancy comparable with the normal population, and there is no justification to expose these cases to any potentially harmful antileukemic therapy [5–7].

However, there has still been a debate, whether cases with a more aggressive disease course could benefit from earlier treatment, in particular with the recent advent of targeted drugs. To date, reported trials that address the role of immediate therapy at an early disease stage have only tested single-agent chemotherapies (i.e., chlorambucil and fludarabine), but no modern treatment options, such as combined chemoimmunotherapy or novel small-molecule inhibitors.

The study presented here (named “CLL7” trial) was aimed at testing whether chemoimmunotherapy with fludarabine, cyclophosphamide, and rituximab (FCR) would improve the outcome of patients with unfavorable prognosis when administered at an early stage. FCR has been the first regimen to prolong survival of advanced-stage CLL, and represents a standard of care option for first-line treatment of physically fit CLL patients [8–12]. We present data of a German–French collaborative phase 3 trial that compared early FCR therapy versus “watch-and-wait” in Binet A patients with the categorized high-risk CLL disease. We implemented an advanced four-parameter risk stratification system, including genetic disease features to prospectively segregate cases with the Binet A high-risk CLL from those with the low-risk disease, and to direct their therapeutic management in a randomized fashion.

## Methods

### Trial design and participants

A prospective randomized phase 3 trial (CLL7) was collaboratively conducted by the German CLL study group (GCLLSG) and the French Cooperative Group on CLL (FCGCLL). Patients with early-stage CLL were registered at 69 sites in Germany, Austria, and Switzerland, and 25 sites in France, in case the following main inclusion criteria had been fulfilled (Supplementary Table 1): diagnosis of CLL according to NCI-working group criteria [13], established not earlier than 12 months prior to registration, Binet stage A disease, no prior treatment, age ≥ 18 years, and Eastern Cooperative Oncology Group performance status 0–2. Patients with clinically evident autoimmune cytopenias, active second malignancies or infections, long-term use of steroids, or other severe medical illnesses or organ dysfunctions were not eligible. All patients provided written informed consent before registration. The trial was conducted according to the Declaration of Helsinki, and approved by ethical review boards responsible for each of the participating centers. It was registered at the US National Institute of Health (NCT00275054) and the EU clinical trial database (EudraCT 2005-003018-14).

### Risk stratification and randomization

After registration, the following risk parameters were assessed in central laboratories of the GCLLSG and FCGCLL according to standard protocols: serum thymidine kinase (TK) levels, the mutation status of the immunoglobulin heavy-chain variable region genes (IGHV), and recurrent chromosomal abnormalities by fluorescence in situ hybridization. The lymphocyte doubling time (LDT) was calculated by regression curve analysis from a minimum of three lymphocyte counts obtained in at least 4-week intervals within 6 months before registration. Risk factor results were collected at the German and French biometry centers, respectively (Institute for Medical Statistics and Epidemiology (IMSE), Technical University of Munich, Germany; Department de Biostatistiques et Informatique Medicale, Hôpital Saint Louis, Paris, France), where for each patient the final risk evaluation and stratification/randomization procedures were performed. Patients with at least two of four adverse prognostic markers present (TK > 10 U/L, LDT < 12 months, IGHV unmutated, or deletion (del) in chromosome 11q or 17p, or trisomy 12) were categorized as high-risk (Hi) patients, while patients with <2 of these markers present were categorized as low risk (Lo). High-risk patients were one-to-one randomized to either receive FCR chemoimmunotherapy (Hi-FCR) or being observed (Hi-W&W) using a previously generated randomization list (IMSE). The randomization was balanced by the use of randomly permuted blocks with a block size of four, and was stratified according to country and number of adverse prognostic markers. Low-risk patients were only assigned to clinical observation (Lo-W&W).

### Patient treatment and procedures

Patients randomized to the Hi-FCR arm were assigned to receive a maximum of six cycles of intravenous FCR, given in 28-day intervals. Fludarabine (25 mg/m^2^) and cyclophosphamide (250 mg/m^2^) were administered on day 1–3 of each cycle. Rituximab was given at 375 mg/m^2^ on day 0 of cycle 1, and at 500 mg/m^2^ on day 1 of cycles 2–6. According to the protocol, the prophylactic use of growth factors was left to the discretion of the local investigator. In case of grades 3–4 neutropenia with signs of a concurrent infection, the administration of G-CSF was mandatory per protocol. Anti-infective prophylaxis with trimethoprim/sulfmethoxazole was recommended from day 1 until the end of 2 months after the last dose of the last cycle. Additional details on parental drug administration, concomitant medication, and dose reduction rules are described in Supplementary Methods.

Baseline disease assessment included physical examination, ECOG performance status, assessment of B symptoms and comorbidity, imaging of disease manifestations via ultrasound or computed tomography (CT), laboratory assessments from peripheral blood (PB) including parameters routinely assessed prior to the administration of cytoreductive therapies, serum beta-2-microglobulin, and lymphocyte immunophenotyping. Patients underwent baseline and follow-up disease assessments at month 4 (interim staging after three cycles of therapy, Hi-FCR only), month 8 (final staging after therapy), and 12, in 6-month intervals between months 12 and 60, and once per year thereafter. Response assessment after FCR therapy included routine clinical and laboratory assessments, radiographic imaging of CLL manifestations (used method at the discretion of the local investigator), and flow cytometry for minimal residual disease (MRD) assessment. The latter was performed using four-color flow cytometry for the German and six-color flow cytometry for the French cohort. For further details refer to Supplementary Methods [14, 15]. A uniform threshold was applied to define MRD negativity as less than one detected CLL cell per 10,000 leukocytes analyzed per flow cytometry. After treatment completion, a bone marrow (BM) aspirate/biopsy was recommended per protocol in case the patient achieved a complete remission (CR).

### Outcomes

The primary objective of the study was to compare the efficacy of early versus deferred FCR in Binet stage A patients at high risk for disease progression. The secondary objective was to prospectively validate the prognostic value of the above-mentioned four-parameter risk stratification system for Binet A patients. The primary endpoint was event-free survival (EFS), considering progression, treatment, or death as events. Among the secondary endpoints were overall response rate (ORR), overall survival (OS), progression-free survival (PFS), adverse events related to treatment, molecular response, response duration, and time to (re)treatment (TTT). The toxicity of FCR treatment was determined according to the Common Terminology Criteria (CTC) for Adverse Events version 3.0. The response status after FCR therapy and disease status during follow-up was evaluated according to the NCI-working group criteria [13].

### Statistical analysis

Details on the sample size computation for this study, data responsibilities, and data sharing are described in Supplementary Methods. The primary analysis was a two-sided log-rank test that was stratified by country and number of risk factors in a second step to confirm the results. Time-to-event endpoints were estimated according to the Kaplan–Meier method. Survival curves were compared using nonstratified log-rank tests. Hazard ratios (HR), including 95% confidence intervals (CI), were calculated by Cox regression analysis under the assumption of proportional hazards. Exploratory post hoc subgroup analyses were done considering MRD status, IGHV mutational status, and cytogenetic categories. All tests were two sided, and a *p* value < 0.05 was considered significant. Adjustments for multiple testing were not done. Safety analyses were restricted to patients from the intention-to-treat population who received at least one dose of one component of the study treatment (safety population). ORR was calculated based on both the intention-to-treat and on the safety population. Statistical analyses were performed using SPSS v23 (SPSS, Chicago/IL, USA).

## Results

### Study population

**Fig. 1:**
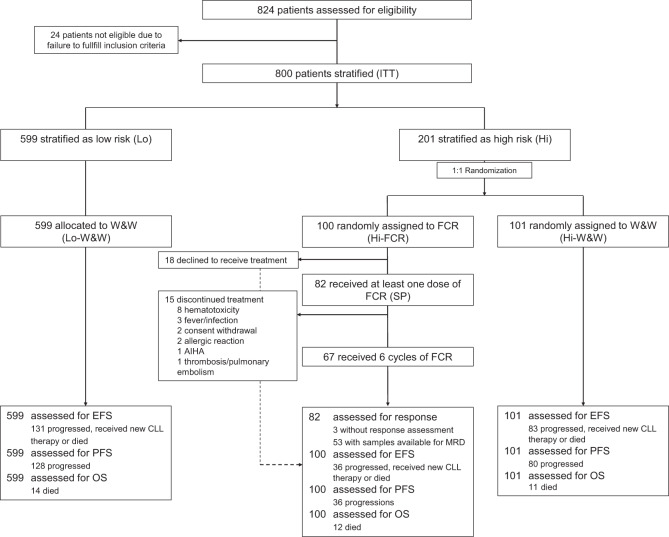
Trial flow diagram illustrating patient assessment and allocation within the CLL7 study. AIHA autoimmune hemolytic anemia, EFS event-free survival, FCR fludarabine, cyclophosphamide, and rituximab, PFS progression-free survival, OS overall survival, W&W watch and wait.

Between 2005 and 2010, a total of 824 patients were registered for the CLL7 study, 423 in 69 GCLLSG centers in Germany (51.3%), Austria, and Switzerland, and 401 (48.7%) in 25 centers of the FCGCLL in France. After exclusion of patients, who did not fulfill the study requirements, and completion of risk assessment, 800 patients (ITT population), aged 27–81 years, were stratified into 201 high-risk (25.1%) and 599 low-risk (Lo-W&W) patients (74.9%) (Fig. 1). The median time from registration to risk stratification was 3 months (0–29.1 months). One hundred and one high-risk patients were randomized to the observation arm (Hi-W&W), while the remaining 100 patients were allocated to receive early FCR (Hi-FCR). Both high-risk arms were well balanced with respect to country, age, comorbidity, ECOG status, white blood count (WBC), IGHV mutation status, trisomy 12, and del(17p) (Table 1). There was an imbalance in the prevalence of elevated TK, short LDT, male sex (each more common in Hi-FCR), and del(11q) (more common in Hi-W&W) between the two high-risk cohorts. B symptoms and lymphadenopathy as signs of a more aggressive disease course were more common in high-risk than in low-risk patients.

**Table 1:** Baseline characteristics (intention-to-treat population).

Parameter	High risk		Low risk	Total *N* (%)
	Hi-FCR *N* (%)	Hi-W&W *N* (%)	Lo-W&W *N* (%)	
All patients	100	101	599	800
Germany	59 (59.0)	60 (59.4)	295 (49.2)	414 (51.8)
France	41 (41.0)	41 (40.6)	304 (50.8)	386 (48.3)
Median age (range)	58 (33–77)	60 (40–81)	59 (27–81)	59 (27–81)
Age ≤ 60 years	58 (58.0)	54 (53.5)	335 (55.9)	447 (55.9)
Age > 70 years	10 (10.0)	15 (14.5)	59 (9.8)	84 (10.5)
Male sex	69 (69.0)	78 (77.2)	366 (61.1)	513 (64.1)
≥1 comorbidity (CIRS^a^, *N* = 765)	54 (54.0)	61 (60.4)	354 (62.8)	469 (61.3)
>6 comorbidities (CIRS^a^, *N* = 765)	2 (2.0)	4 (4.0)	6 (1.1)	12 (1.6)
ECOG^b^ 0–1 (*N* = 751)	94 (98.9)	100 (100.0)	554 (98.9)	748 (99.1)
B symptoms (*N* = 762)	8 (8.1)	9 (9.1)	25 (4.4)	42 (5.5)
Clinical lymphadenopathy > 1 cm (*N* = 772)	48 (48.0)	49 (48.5)	159 (27.8)	256 (33.2)
Radiologic lymphadenopathy > 1 cm (*N* = 772)	49 (49.0)	41 (40.6)	130 (22.8)	220 (28.5)
Median WBC, ×10E3/µl (range, *N* = 766)	28 (7.2–220.0)	30 (0.1–165.6)	18 (5.8–239.8)	20 (0.1–239.8)
TK > 10 U/L (*N* = 795)	62 (62.0)	52 (52.0)	40 (6.7)	154 (19.4)
LDT < 12 months (*N* = 799)	58 (58.0)	48 (47.5)	71 (11.9)	177 (22.2)
IGHV unmutated (*N* = 783)	81 (81.0)	82 (82.0)	57 (9.8)	220 (28.1)
Cytogenetics (*N* = 795)^c^	100	100	595	795
Trisomy 12 (*n*, %)	25 (25.0)	24 (24.0)	18 (3.0)	67 (8.4)
Del(11q) (*n*, %)	17 (17.0)	35 (35.0)	3 (0.5)	55 (6.9)
Del(17p) (*n*, %)	4 (4.0)	9 (9.0)	5 (0.8)	18 (2.3)
Not del(17p)/del(11q)/trisomy 12	54 (54.0)	32 (32.0)	569 (95.6)	655 (82.4)
Total risk factors (*N* = 799)	100	100	599	799
0	0 (0.0)	0 (0.0)	395 (65.9)	395 (49.4)
1	0 (0.0)	1 (1.0)^d^	202 (33.7)	203 (25.4)
2	58 (58.0)	55 (55.0)	2 (0.3)^d^	115 (14.4)
3	34 (34.0)	36 (36.0)	0 (0.0)	70 (8.8)
4	8 (.0)	8 (8.0)	0 (0.0)	16 (2.0)

^a^Cumulative illness rating scale [32].

^b^ECOG = performance status scale according to the Eastern Cooperative Group [33].

^c^According to Döhner et al. [24].

^d^Three patients were allocated to the incorrect risk stratum according to their risk profile presented here. Two of those cases (one Hi-W&W and one Lo-W&W) were caused by entry/capture errors for assigned risk factors in the database; and, these patients were stratified in the correct risk subset. Only one Lo-W&W patient was truly misstratified as a low-risk case, despite the fact that two risk factors had been found present by central diagnostics.

### Early FCR treatment and safety

Eighty-two percent of 100 Hi-FCR patients received at least one dose of FCR, and were included in the safety analysis (safety population). Eighteen percent patients withdrew their consent for early therapy after randomization and before FCR had been initiated. The median number of administered treatment cycles was 6 (range, 1–6) and 67 patients (81.7%) completed six cycles of study therapy. The documented reasons for discontinued FCR (<6 cycles, 15 patients, 18.3%) were hematotoxicity (6 patients, predominantly neutropenia), fever/infections (2 patients; 1 CMV reactivation, 1 infection of unknown origin), consent withdrawal or allergic exanthema (2 patients each), 1 hospitalization due to rupture of an aortic aneurysm, 1 thrombosis with consecutive pulmonary embolism, and 1 autoimmune hemolytic anemia (AIHA).

In 20 patients (24.4%) at least one study drug was dose reduced >20% in one or more cycles. Most frequently, the doses of fludarabine and cyclophosphamide were reduced (15 cases) due to hematologic toxicity (11 cases with at least one of the following events: 9 leukopenia/neutropenia, 2 thrombocytopenia, 1 cytopenia not further specified, and 1 anemia). Other reasons for dose-reduced FC were febrile infections (two cases), inpatient treatment due to a ruptured aortic aneurysm (one case), a collapse during infusion (one case), or unknown (two cases). Dose-reduced rituximab was given in five patients (twice unintentionally by missing the rituximab dose increase at cycle 2, in two cases for unknown reasons, and in one case due to an event of bradycardia).

Overall, 203 grade 3–5 adverse events in 61 patients (74.4% of safety population) were reported. In addition, there were 18 events documented in five patients, without sufficient information (including missing CTC grade) available. One hundred and twenty-five of those 203 events (86.2%) were categorized as at least possibly related to the study treatment by the local investigator. The three most common categories were hematotoxicity (50 patients, 61.0% of safety population; most frequently leukopenia/neutropenia), infections (18 patients, 22.0%), and metabolic/laboratory events (5 patients, 6.1%; most frequently elevated liver enzymes) (Table 2). Recurrent types of infections were respiratory tract infections (seven patients, 8.5% of the safety population), fever/infections of unknown origin (three patients, 3.7%), herpes zoster reactivations (three patients, 3.7%), and catheter-related infections (two patients, 2.4%). Use of growth factor support with G-CSF was documented in 25 out of 82 FCR treated patients (30.5%).

**Table 2:** CTC grade ≥ 3 adverse events (AE) in patients treated with early FCR (safety population, *n* = 82).

	Total (CTC 3–5) *n* (%)	CTC grade 3 *n* (%)	CTC grade 4 *n* (%)	CTC grade 5 *n* (%)	Unknown grade *n* (%)
*N* patients with at least one ≥ CTC grade 3 AE	61 (74.4)	28 (34.1)	29 (35.4)	4 (4.9)	5 (6.1)
CTC AE category^**a**^					
Blood/bone marrow	50 (61.0)	23 (28.0)	26 (31.7)	1 (1.2)	0 (0.0)
Neutropenia	37 (45.1)	15 (18.3)	22 (26.8)	0 (0.0)	0 (0.0)
Leukopenia	25 (30.5)	19 (23.2)	6 (7.3)	0 (0.0)	0 (0.0)
Thrombocytopenia	5 (6.1)	1 (1.2)	4 (4.9)	0 (0.0)	0 (0.0)
Anemia	4 (4.9)	4 (4.9)	0 (0.0)	0 (0.0)	0 (0.0)
Cytopenia	1 (1.2)	1 (1.2)	0 (0.0)	0 (0.0)	0 (0.0)
Hemolysis	1 (1.2)	0 (0.0)	0 (0.0)	1 (1.2)	0 (0.0)
Infection	18 (22.0)	16 (19.5)	1 (1.2)	1 (1.2)	0 (0.0)
Respiratory tract infection	7 (8.5)	7 (8.5)	0 (0.0)	0 (0.0)	0 (0.0)
Fever/infection of unknown origin	3 (3.7)	3 (3.7)	0 (0.0)	0 (0.0)	0 (0.0)
Herpes zoster	3 (3.7)	3 (3.7)	0 (0.0)	0 (0.0)	0 (0.0)
Catheter-related infection	2 (2.4)	2 (2.4)	0 (0.0)	0 (0.0)	0 (0.0)
CMV reactivation	1 (1.2)	1 (1.2)	0 (0.0)	0 (0.0)	0 (0.0)
Bursitis	1 (1.2)	1 (1.2)	0 (0.0)	0 (0.0)	0 (0.0)
Candida esophagitis	1 (1.2)	1 (1.2)	0 (0.0)	0 (0.0)	0 (0.0)
Hepatitis B	1 (1.2)	0 (0.0)	1 (1.2)	0 (0.0)	0 (0.0)
Sepsis	1 (1.2)	1 (1.2)	0 (0.0)	0 (0.0)	0 (0.0)
Splondylodiscitis	1 (1.2)	1 (1.2)	0 (0.0)	0 (0.0)	0 (0.0)
Viral encephalititis	1 (1.2)	0 (0.0)	0 (0.0)	1 (1.2)	0 (0.0)
Metabolic/laboratory	5 (6.1)	4 (4.9)	1 (1.2)	0 (0.0)	0 (0.0)
Elevated GOT, GPT, or GGT	4 (4.9)	3 (3.7)	1 (1.2)	0 (0.0)	0 (0.0)
Hyperglycemia	1 (1.2)	1 (1.2)	0 (0.0)	0 (0.0)	0 (0.0)
Gastrointestinal	3 (3.7)	2 (2.4)	1 (1.2)	0 (0.0)	0 (0.0)
Nausea/vomiting	1 (1.2)	1 (1.2)	0 (0.0)	0 (0.0)	0 (0.0)
Obstipation	1 (1.2)	1 (1.2)	0 (0.0)	0 (0.0)	0 (0.0)
Diarrhea	1 (1.2)	0 (0.0)	1 (1.2)	0 (0.0)	0 (0.0)
Vascular	4 (4.9)	4 (4.9)	0 (0.0)	0 (0.0)	0 (0.0)
Thrombosis	2 (2.4)	2 (2.4)	0 (0.0)	0 (0.0)	0 (0.0)
Thrombosis with consecutive pulmonary embolism	2 (2.4)	2 (2.4)	0 (0.0)	0 (0.0)	0 (0.0)
Ruptured aortic aneurysm	1 (1.2)	1 (1.2)	0 (0.0)	0 (0.0)	0 (0.0)
Neurology	2 (2.4)	2 (2.4)	0 (0.0)	0 (0.0)	0 (0.0)
Depression	1 (1.2)	1 (1.2)	0 (0.0)	0 (0.0)	0 (0.0)
Orthostasis	1 (1.2)	1 (1.2)	0 (0.0)	0 (0.0)	0 (0.0)
Pain	2 (2.4)	2 (2.4)	0 (0.0)	0 (0.0)	0 (0.0)
Herniated disc/radiculopathy	2 (2.4)	2 (2.4)	0 (0.0)	0 (0.0)	0 (0.0)
Dermatology/skin	2 (2.4)	1 (1.2)	1 (1.2)	0 (0.0)	0 (0.0)
Exanthema	2 (2.4)	1 (1.2)	1 (1.2)	0 (0.0)	0 (0.0)
Circulatory symptoms/arrhythmia	2 (2.4)	1 (1.2)	1 (1.2)	0 (0.0)	0 (0.0)
Circulatory symptoms/arrhythmia	2 (2.4)	1 (1.2)	1 (1.2)	0 (0.0)	0 (0.0)
Cardiac arrhythmia	1 (1.2)	0 (0.0)	1 (1.2)	0 (0.0)	0 (0.0)
Circulatory symptoms/arrythmia	1 (1.2)	0 (0.0)	1 (1.2)	0 (0.0)	0 (0.0)
Cardiac general	1 (1.2)	1 (1.2)	0 (0.0)	0 (0.0)	0 (0.0)
Circulatory symptoms/arrythmia	1 (1.2)	1 (1.2)	0 (0.0)	0 (0.0)	0 (0.0)
Constitutional symptoms	1 (1.2)	1 (1.2)	0 (0.0)	0 (0.0)	0 (0.0)
Constitutional symptoms	1 (1.2)	1 (1.2)	0 (0.0)	0 (0.0)	0 (0.0)
Musculoskeletal/soft tissue	1 (1.2)	1 (1.2)	0 (0.0)	0 (0.0)	0 (0.0)
Bone fracture (nonpathologic)	1 (1.2)	1 (1.2)	0 (0.0)	0 (0.0)	0 (0.0)
Pulmonary/upper respiratory	1 (1.2)	0 (0.0)	0 (0.0)	1 (1.2)	0 (0.0)
Pulmonary fibrosis	1 (1.2)	0 (0.0)	0 (0.0)	1 (1.2)	0 (0.0)
Renal/genitourinary	1 (1.2)	0 (0.0)	0 (0.0)	1 (1.2)	0 (0.0)
Renal insufficiency	1 (1.2)	0 (0.0)	0 (0.0)	1 (1.2)	0 (0.0)

^a^According to CTC v3.0. Adverse events are shown of CTC grade 3, 4, or 5, which occurred at least in patient per event category.

There were two of total four fatal adverse events during follow-up, documented with a potential relationship to administered FCR: one patient succumbed 9.3 months from stratification to a suspected viral encephalitis (clinical/radiologic diagnosis), which was judged as possibly related to the study therapy. Another patient died 9.8 months from stratification due to a persisting AIHA that had occurred under FCR therapy. Two additional deaths were documented at month 41.8 and 53.2 from stratification with no relationship to study therapy. Causes of death were reported as a pulmonary fibrosis and progressive renal failure in the context of a Richter’s transformation, respectively.

In the overall study, we did not detect an elevated risk of disease transformation, second(ary) malignancies, or AIHA related to an early FCR therapy (Supplementay Table 2). One Hi-FCR patient developed a secondary acute myeloid leukemia (AML) at month 43.4 during treatment with R-CHOP for Richter’s transformation. Two Lo-W&W patients developed an AML at month 10.9 and at month 90.4, respectively. Both patients had received prior FCR for progressive CLL. Ten patients (1.25% of ITT) developed a Richter’s transformation after median 50.1 months (11.6–84.8 months), two in the Hi-FCR arm (2.0%), three in the Hi-W&W arm (3.0%), and five in the Lo-W&W cohort (0.8%).

### Efficacy of early FCR and survival

The overall response to early FCR based on the ITT population according to NCI-working group criteria [13] was 76.0% (76 out of 100 patients allocated to the Hi-FCR arm). Out of 82 patients, who had received at least one dose of FCR (safety population), 76 (92.7%) achieved a remission (Table 3). Twenty-seven patients (32.9%) obtained a BM confirmed CR, 34 patients (41.5%) an at least clinical CR without BM evaluation, and 15 patients (18.3%) obtained a partial remission (PR). In three patients, response assessment was missing, but they had received only one or two cycles of the study therapy, respectively. Three patients (3.8%) had stable disease after therapy. In two of those three cases treatment had been stopped prematurely after one or two cycles due to grade 3 neutropenia and grade 3 febrile neutropenia, respectively. The highest CR rates were achieved in patients who underwent at least three cycles of therapy, were IGHV mutated, or carried a del(11q).

**Table 3:** Response to treatment overall and in post hoc analysis of subgroups.

Patients assessed for response (Hi-FCR)	Overall response *N* (%)	Complete response *N* (%)	Partial response *N* (%)	Stable disease *N* (%)	Not evaluable/available *N* (%)
All patients (ITT, *N* = 100)	76 (76.0)	61 (61.0)	15 (15.0)	3 (3.0)	21 (21.0)^a^
Treated patients (SP, *N* = 82)	76 (92.7)	61 (74.4)	15 (18.3)	3 (3.7)	3 (3.7)
Patients with ≥ 3 cycles of FCR (*N* = 75)	74 (98.7)	61 (81.3)	13 (17.3)	1 (1.3)	0 (0.0)
Two risk factors present (*N* = 58)	44 (75.9)	36 (62.1)	8 (13.8)	1 (1.7)	13 (22.4)
Three risk factors present (*N* = 34)	26 (76.5)	20 (58.8)	6 (17.6)	2 (5.9)	6 (17.6)
Four risk factors present (*N* = 8)	6 (75.0)	5 (62.5)	1 (12.5)	0 (0.0)	2 (25.0)
LDT < 12 months (*N* = 58)	43 (74.1)	38 (65.5)	5 (8.6)	2 (3.4)	13 (22.4)
TK > 10 U/L (*N* = 62)	50 (80.6)	38 (61.3)	12 (19.4)	2 (3.2)	10 (16.1)
IGHV unmutated (*N* = 81)	59 (72.8)	45 (55.6)	14 (17.3)	3 (3.7)	19 (23.5)
IGHV mutated (*N* = 19)	17 (89.5)	16 (84.2)	1 (5.3)	0 (0.0)	2 (10.5)
Trisomy 12^b^ (*N* = 25)	18 (72.0)	16 (64.0)	2 (8.0)	0 (0.0)	7 (28.0)
Del(11q)^b^ (*N* = 17)	15 (88.2)	12 (70.6)	3 (17.6)	0 (0.0)	2 (11.8)
Del(17p)^b^ (*N* = 4)	2 (50.0)	0 (0.0)	2 (50.0)	1 (25.0)	1 (25.0)
No trisomy 12/del(11q)/del(17p) (*N* = 54)	41 (75.9)	33 (61.1)	8 (14.8)	2 (3.7)	11 (20.4)

^a^Includes 18 patients who refused initiation of FCR therapy after stratification/randomization.

^b^According to Döhner et al. [24].

Fifty-three and 28 Hi-FCR patients were available for MRD assessment by four-color flow cytometry from PB and BM, respectively. Forty of 53 patients (75.5%) were MRD negative (≤10^−4^) in PB at the time of final response assessment, 13 patients (24.5) were MRD positive (>10^−4^). In BM, 67.9% (19 out of 28 patients) achieved an MRD-negative remission.

**Fig. 2:**
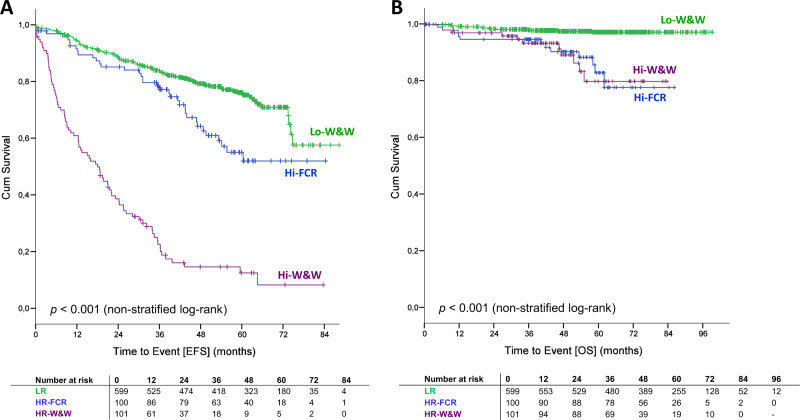
Event-free survival (EFS) and overall survival (OS) according to risk stratification/randomization (ITT). **a** EFS from stratification. **b** OS from stratification. Hi-FCR high-risk CLL treated with early FCR, Hi-W&W high-risk CLL under observation, and Lo-W&W low-risk CLL under observation (watch and wait).

**Fig. 3:**
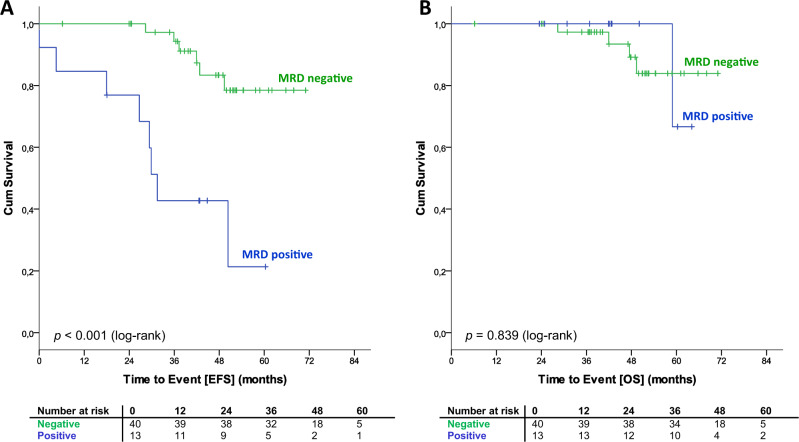
Event-free survival (EFS) and overall survival (OS) according to MRD status in peripheral blood. **a** EFS from MRD landmark (final response assessment/MRD evaluation). **b** OS from MRD landmark (final response assessment/MRD evaluation). MRD minimal residual disease. For this calculation, the MRD status at the final restaging was considered. MRD negative < 10^−4^; positive ≥ 10^−4^ detected CLL cells per leukocytes, according to MRD-flow cytometry. For MRD-results from bone marrow please refer to Supplementary Figs. 1 and 2.

The primary endpoint, EFS, was significantly prolonged in high-risk patients treated with an early FCR (Hi-FCR) versus deferred treatment according to the current standard of care (Hi-W&W). After a median follow-up of 55.6 months (range 0–99.2 months), only 36 patients (36.0%) in the Hi-FCR arm had progressed, received new CLL therapy, or died, compared with 83 patients (82.2%) in the Hi-W&W arm (median EFS not reached in Hi-FCR vs. 18.5 months in Hi-W&W, HR 0.22, 95% CI 0.15–0.33, *p* < 0.001 for stratified and nonstratified log-rank test) (Fig. 2a). High-risk patients with a MRD-negative response to early FCR in PB significantly benefited from the quality of remission with regard to EFS compared with patients with an MRD-positive response (landmark analysis, median EFS not reached versus 41.2 months, log-rank *p* < 0.001, HR 10.68, 95%CI 3.51–32.55, Fig. 3a; for MRD from BM refer to Supplementary Fig. 1). Twelve Hi-FCR and 11 Hi-W&W patients had died. In both studies, arms major causes of death were infections and progressive disease including Richter’s transformation (Supplementary Table 3). There was no significant OS benefit for high-risk patients receiving early versus deferred FCR (5-year OS 82.9% in Hi-FCR vs. 79.9% in Hi-W&W, HR 0.93, 95% CI 0.41–0.22, *p* = 0.864, Figs. 2b and 3b, Supplementary Fig. 2).

**Fig. 4:**
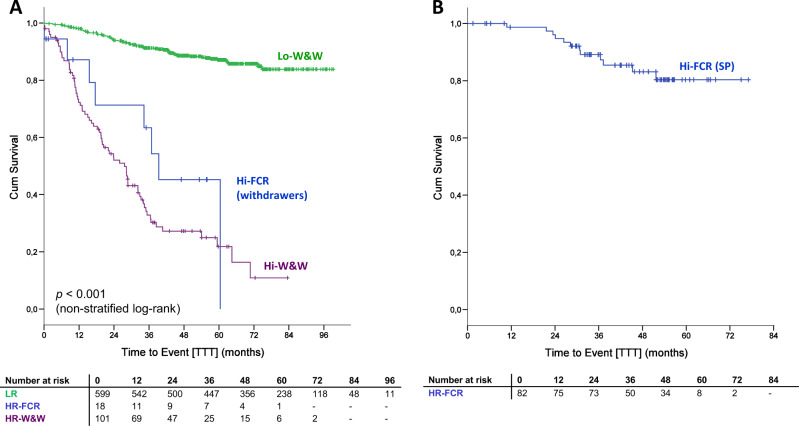
Time to (re)treatment (TTT) according to risk stratification/randomization and treatment status per protocol. **a** Time to first treatment (TTT) in Hi- and Lo-risk patient categories, considering also Hi-FCR patients, who had withdrawn their consent for early FCR after trial inclusion. **b** Time to re-treatment in Hi-FCR patients, who actually underwent early FCR therapy according to the protocol (SP safety population).

After 5 years from the last dose of study medication, 80.4% of Hi-FCR patients (safety population) had not received any further treatment for CLL (median TTT not reached, Fig. 4b), compared with 21.8% of patients in the Hi-W&W arm (Fig. 4a).

Our risk stratification system successfully segregated patients with differential prognosis. High-risk patients with or without early FCR therapy (Hi-FCR/Hi-W&W) demonstrated a significantly shorter EFS, PFS, and OS than patients categorized with the low-risk disease (Fig. 2a, b, Supplementary Fig. 3). Briefly, patients assigned to Hi-W&W had a 8.0 times higher risk of progression, treatment or death (HR 8.02, 95%CI 6.04–10.65, *p* < 0.001), while Hi-FCR patients had a 1.8 times higher risk (HR 1.82, 95% CI 1.26–2.63, *p* = 0.002) of progression, treatment or death, compared with the Lo-W&W cohort. Corresponding 5-year EFS rates were 12.6%, 55.2%, and 77.1% in Hi-W&W, Hi-FCR, and Lo-W&W patients, respectively. In total, 87.1% of Lo-W&W patients were without treatment at 5 years from stratification (median time to first-line treatment 27.6 months in Hi-W&W vs. not reached in Lo-W&W, HR 11.62, 95% CI 8.23–16.39, *p* < 0.001). Patients with the low-risk disease demonstrated an excellent survival at 97.2% 5 years from stratification (HR Hi-FCR/W&W vs. Lo-W&W 5.82, 95% CI 2.98–11.36, *p* < 0.001) (Fig. 2b).

## Discussion

We present data of a phase 3 trial (CLL7), which successfully implemented molecular genetic disease characteristics into a risk-tailored treatment allocation strategy for patients with stage Binet A CLL. Twenty-five percent of our ITT study population exhibited a “high risk” disease type according to our four-factor risk assessment, and these patients clearly segregated from the low-risk group with regard to all time-to-event parameters investigated, i.e., illustrated by EFS, PFS (Supplementary Fig. 3), and OS.

All four risk parameters used for our study design were chosen due to their confirmed value as prognostic factors for PFS/OS in multivariate analyses performed in the first 147 patients registered in the preceding CLL1 trial (phase 3 comparison of fludarabine vs. W&W in Binet A CLL) in 2004 [2]. In particular, we found serum levels of the TK (cutoff 10 U/L) rather than beta-2-microglobulin (3.5 mg/L) as a preferred independent prognostic factor for time-to-event outcome in our test set analysis, and therefore implemented serum TK in our study design [16]. The parameter LDT reflects the disease dynamics, and is recommended by current guidelines to determine the right time a patient requires therapy [17]. Particularly at an early disease stage, an LDT < 12 months has been identified as an independent indicator of an unfavorable prognosis [18–20]. Although easily assessable in clinical practice, the parameter is not commonly documented in large trial datasets, and therefore not considered in the latest CLL scoring systems, such as the CLL-IPI [21–23].

The scientific background to include trisomy 12 as a risk factor in our stratification approach was formed by the hierarchical model, developed by Döhner et al. before this study was designed [24]. Recent long-term follow-up data in FCR studies, however, demonstrated that patients with trisomy 12 have a particularly favorable PFS/OS after FCR, when treated at an advanced disease stage [9, 25, 26]. Thus, in retrospect it might have been specifically difficult to achieve further improvement for this patient population with our early treatment strategy (Supplementary Figs. 4 and 14).

A comparative analysis of our risk stratification and the CLL-IPI as a current standard risk assessment in CLL is included as Supplementary Table 4. It indicates that the CLL7 stratification between low-risk and high-risk subsets correponds to a segregation between CLL-IPI low risk versus CLL-IPI intermediate/high/very high risk in the majority of cases.

The data presented here demonstrate that an early application of FCR was able to postpone events of disease progression and the need of therapy in stage Binet A high-risk CLL, but despite this effect, there was no OS benefit in the long run.

FCR was highly effective in reducing the tumor load in treated patients, as demonstrated by a high OR and CR rate. Moreover, while the significance of the MRD data set is limited by a relatively low number of assessments, the frequency of achieved MRD negativity (PB: 75.5%, BM: 67.9%) compares favorably to the respective data from the FCR arm of the CLL8 trial (63% and 44%, respectively) [27]. Patients who achieved a MRD-negative status (at a threshold of 10^−4^ in PB) appeared to enjoy a better prognosis (median EFS not reached) than previously reported for MRD-negative patients with active disease treated within the CLL8 trial (median PFS 64 months). These findings underline not only the important ability but also potentially higher likelihood of disease-eradicating activity by treatment regimens applied at an early disease stage.

The shortcomings of this study might be the primary endpoint EFS from stratification may be criticized for not considering the difference in the disease load in early treated versus observed patients, and hence, for implementing an upfront advantage or disadvantage, respectively, in the risk of progression. It should be considered that this study was initiated at a time when the clinical experience with FCR, used at an advanced disease stage, was still limited to make projections on outcome for a study design like ours. We preferred to choose a primary endpoint, which commences at trial outset for all patients, most independent from other dynamic variables, and which allows a study design realistic to be accrued.

Not all patients in the Hi-W&W arm did receive FCR as a deferred frontline therapy. Per protocol, the use of FCR was recommended, in case Hi-W&W patients were in need of therapy. According to collected data on the choice of first-line therapy in the Hi-W&W arm (available in 70 patients, Supplementary Table 5), the use of anti-CD20 treatment was a common choice made for first-line therapy in the Hi-W&W arm, but also use of less efficacious treatments (i.e., R-CHOP, obinutuzumab + chlorambucil, various monotherapies) were given. In addition, the application of new oral kinase/small-molecule inhibitors at later disease stages in the overall high-risk population might have influenced the survival data as they are.

It could be argued that an elevated risk to die from treatment-related early or late toxicity might have mitigated any survival benefits in the Hi-FCR arm. In comparison to other studies investigating frontline FCR at an advanced disease stage, our study did not clearly detect a significantly higher or unexpected toxicity of FCR, when administered at an early stage. For example, the documented rate of CTC grade 3/4 hematotoxicity after deferred FCR was 56% in the CLL8 trial (phase 3 registration study for FCR versus fludarabine plus cyclophosphamide (FC)) [9]. In the FCR arm of the CLL10 study (phase 3 study on FCR versus BR) [28], grade 3/4 neutropenia occurred in 84% of patients, the overall rate of patients with grade 3 hematological events was 21% and 69% for grade 4, respectively. We observed grade 3/4 infections in 22% of treated patients in the Hi-FCR arm compared with 25% of patients treated with FCR in CLL8, and 35% (grade 3) and 3% (grade 4) of patients treated with FCR in CLL10. The use of growth factors was not generally recommended in all of these protocols and not equally documented for a head-to-head comparison. Further, the causes of death documented in both high-risk arms of our study—predominantly progressive CLL disease and infections—did reveal an increased mortality by late adverse treatment effects. Although a direct comparison of toxicity rates between different trials has to be interpreted with caution, these data allow the conclusion, that the tolerability of FCR in our study was comparable to what has been experienced with its use in advanced-stage CLL. A mandatory use of growth factors like G-CSF might have been adequate to limit the rate of neutropenia and the associated risk of infections.

To rule out a particular hazard of an early FCR in a distinct molecular subset of patients we also compared time-to-event outcome according to the IGHV mutation status, and in cytogenetic subsets [24] (Supplementary Figs. 4–15). No particular benefit or disadvantage of early versus late therapy could be detected in these subgroups with respect to EFS and OS. Although not statistically significant due to low patient numbers, there was a particular adverse disease course in three of four early treated patients with del(17p), who died within 12.2 months from stratification (Supplementary Fig. 13). The causes of death were persisting AIHA, a cerebral stroke, and hemophagocytosis/infectious complications after allogeneic stem cell transplant, respectively.

Molecular genetic studies in advanced CLL have revealed a high level of clonal heterogeneity and ongoing genetic evolution of CLL cells throughout the disease course and in particular under applied treatment pressure [29, 30]. Clinically, clonal evolution might have become evident in the Hi-FCR arm of our trial with lower remission rates or response durations after second-line therapies. These data were not the focus of this trial or analysis. However, those considerations warrant careful monitoring of molecular alterations evolving under ongoing treatment pressure, and their consequences on sequential treatment outcome in future studies in early-stage CLL.

In conclusion, FCR therapy is feasible in Binet A stage CLL and extends EFS and PFS in patients with high-risk disease. As a caveat of early FCR we observed possibly treatment-related deaths in 2.4% of treated patients. In accordance with previous treatment studies in an early-stage CLL, our trial does not provide any evidence that the significant improvement of EFS in this patient population translates into a survival benefit. Therefore, “watch & wait” after diagnosis, until “active disease” criteria [31] are met, remains the standard of care, irrespective of unfavorable prognostic features. Ongoing and future studies may elucidate, whether the immediate use of such targeted and potentially disease-eradicating therapies (i.e., venetoclax combinations), will be able to overcome adverse disease courses (particularly for patients with del(17p)), and to displace the current standard of care “watch & wait” [11].

### Supplementary information

Supplemental files
