# Supplementary material for: Early treatment with FCR versus watch and wait in patients with stage Binet A high-risk chronic lymphocytic leukemia (CLL): a randomized phase 3 trial
Source: Leukemia. 2020 Feb 18;34(8):2038–50. doi: 10.1038/s41375-020-0747-7 (PMC7387319; doi:10.1038/s41375-020-0747-7)
Supplement: Supplementary file 1 — Supplemental files [file 41375_2020_747_MOESM1_ESM.docx]

**Supplemental Files**

**Content: Page**

**Suppl. Table 1:** CLL7 inclusion and exclusion criteria 2

**Suppl. Methods:** Sample size calculation, secondary endpoints 3

Parenteral drug administration 3

Concomitant medication 4

Dose reduction rules 4

Minimal residual disease (MRD) assessment 4

Responsibilities and data sharing 5

**Suppl. Table 2:** Second malignancies (all cohorts) 6

**Suppl. Figure 1:** EFS in MRD-negative versus –positive patients

as detected in bone marrow 8

**Suppl. Table 3:** Causes of death in high-risk cohorts (Hi-FCR & Hi-W&W) 9

**Suppl. Figure 2:** OS in MRD-negative versus –positive patients

as detected in bone marrow 10

**Suppl. Figure 3:** PFS (comparison of all study cohorts based on ITT) 11

**Suppl. Figure 4:** EFS for patients with trisomy12 (high-risk cohorts) 12

**Suppl. Figure 5:** OS for *IGHV* mutated patients (high-risk cohorts) 13

**Suppl. Table 4:** Distribution of CLL-IPI risk in the study cohorts 14

**Suppl. Table 5:** First-line therapies administered in Hi-W&W 15

**Suppl. Figure 6:** EFS for *IGHV* mutated patients (high-risk cohorts) 17

**Suppl. Figure 7:** EFS for *IGHV* unmutated patients (high-risk cohorts) 18

**Suppl. Figure 8:** EFS for patients with del(11q) (high-risk cohorts) 19

**Suppl. Figure 9:** EFS for patients with del(17p) (high-risk cohorts) 20

**Suppl. Figure 10:** EFS for patients without del(11q)/del(17p)/trisomy 12

(high-risk cohorts) 21

**Suppl. Figure 11:** OS for *IGHV* unmutated patients (high-risk cohorts) 22

**Suppl. Figure 12:** OS for patients with del(11q) (high-risk cohorts) 23

**Suppl. Figure 13:** OS for patients with del(17p) (high-risk cohorts) 24

**Suppl. Figure 14:** OS for patients with trisomy 12 (high-risk cohorts) 25

**Suppl. Figure 15:** OS for patients without del(11q)/del(17p)/trisomy 12 (high-risk cohorts) 26

**Supplemental Table 1: CLL7 inclusion and exclusion criteria**

|  | **Inclusion criteria** |
| --- | --- |
| 1 | Established diagnosis of B-CLL according to NCI criteria. |
| 2 | Binet stage A. |
| 3 | First diagnosis within 12 months before inclusion in the study. |
| 4 | Start of therapy possible within 28 days after completed risk stratification/ randomization |
| 5 | No prior chemotherapy, radiation or antibody treatment. |
| 6 | Age ≥ 18 years. |
| 7 | Life expectancy > 6 months. |
| 8 | ECOG performance status 0 – 2. |
| 9 | Written informed consent of patient and treating physician available. |
| 10 | All parameters for risk stratification present. |
| 11 | Willingness to accept contraception if randomized to cohort I for the duration of therapy and 12 months thereafter. |
| 12 | Negative serum pregnancy test one week prior to treatment for premenopausal women. |
| 13 | Ability to understand the protocol. |
| 14 | Possibility of (longterm) follow-up. |
|  | |
|  | **Exclusion criteria** |
| 1 | Age < 18 years. |
| 2 | ECOG performance status > 2. |
| 3 | Clinically apparent autoimmune cytopenia, in particular antiglobulin test positive hemolytic anemia (a positive antiglobulin test without anemia is not an exclusion criterion). |
| 4 | Active secondary malignancy or chemotherapy/radiotherapy for any neoplastic disease other than B-CLL prior to the study. |
| 5 | Medical condition requiring prolonged (estimated to be more than one month) use of oral corticosteroids. |
| 6 | History of anaphylactic reaction following exposure to humanized monoclonal antibodies. |
| 7 | Patients with active bacterial, viral, or fungal infection. |
| 8 | Known infection with HIV, Hepatitis B, or C. |
| 9 | Treatment with any other investigational agent or participating in another trial within 30 days prior to entering this study. |
| 10 | Pregnancy and/or nursing. |
| 11 | Concurrent severe diseases which exclude the administration of therapy:  o Heart insufficiency NYHA grade III/IV, LEVF < 50% and or RF < 30%, myocardial infarction within the past 6 months prior to study.  o Severe chronic obstructive lung disease with hypoxemia.  o Severe diabetes mellitus.  o Hypertension difficult to control.  o Impaired renal function with creatinine clearance < 70 ml/min according to the formula of Cockroft and Gault.  o Serum bilirubin > 2 x ULN (upper level of normal value).  o Cerebral dysfunction or any other coexisting medical or psychological condition that would preclude participation in the required study procedures. |
| 12 | Transformation in an aggressive B cell malignancy (i.e. diffuse large cell lymphoma, Richter’s syndrome or prolymphocytic leukemia. |

**Supplemental Methods:**

**Sample size calculation, secondary endpoints**

The sample size calculation for this study was based on assumptions obtained in the CLL1 protocol of the GCLLSG (phase 3 comparison of fludarabine versus watch & wait in high-risk CLL)^[[1]](#footnote-1)^: We hypothesized that early treatment with FCR (Hi-FCR) is superior to observation and deferral of CLL treatment (Hi-W&W), and increases the expected EFS from 50% to 70% after 3 years. The proportional fraction of high-risk patients, as defined in our study, was estimated at 25%. Further, a group sequential design according to O’Brien and Fleming, as implemented by Lan and DeMets, using an α-spending function to allow for one interim and one final analysis was applied. With a sample size of 77 in each high-risk subset, a total number of 57 EFS events was required to reject the nullhypothesis (equality of the two study arms) at an 80% power (adjusted alpha 0.048, one sided). 154 patients needed to be randomized and a total of 600 patients to be enrolled with these assumptions. As the actual study recruitment was unexpectedly delayed (5 years instead of planned 24 months) and internal safety monitoring did not reveal any objections against early FCR therapy, only one primary analysis of all data obtained in the intention-to-treat population (ITT) was performed.

Analyses regarding response duration, time to progression to Binet stages B and C, quality of life, and pharmacoeconomic analyses were not part of this analysis and will be performed and presented separately.

**Parental drug administration**

Parental drug administration was performed as outlined in the study protocol. Briefly, the study medication was given intravenously (peripheral line) in six treatment cycles, each of 28 days duration. Each cycle included fludarabine (25 mg/m2, e.g. Fludarabinphosphat-GRY®) and cyclophosphamide (250 mg/m2, e.g. Endoxan®), administered on day 1 to 3 of each cycle. Rituximab was given at 375 mg/m2 on day 0 of cycle 1, and at 500 mg/m2 on day 1 of cycle 2 to 6. Only the study medication rituximab (e.g. Mabthera®) was provided by the Roche Pharma AG, Grenzach, Germany/Boulogne-Billancourt Cedex, France. All drugs were diluted and stored according to the manufacturer recommendations. The antibody-infusion with rituximab was recommended to be started at an initial rate of 50 mg/hour during the first hour of administration with concomitant monitoring of vital signs (blood pressure, pulse, respiration, and temperature. If no side effects were observed, dose rate had to be escalated in 50 mg/hour increments every 30 minutes to a maximum infusion rate of 300 mg/hour. For the second and subsequent cycles rituximab was allowed to be administered more rapidly in case it was well tolerated by the patient. In case of fever >38.5°C, mild/moderate rigors, signs of mucosal congetion/edema or drop in systolic blood pressure >30 mmHg, or other side effects, a 50% decrease or limitation to a low level (e.g. 50 mg/h) of infusion rate or stop was recommended.

**Concomitant medication**

Premedication with acetaminophen, antihistaminics, antiemetics (i.e. with 5-HT3-antagonists) and prednisone/prednisolone (prior to first rituximab infusion and/or in case of increased risk of tumor lysis/cytokine release, i.e. leukocytosis >50 G/L) was recommended. In case of leukocytosis exceeding 100 G/L, additional hydration, urine alkalinisation, sodium bicarbonate and uricostatics were recommened prior to the first dose of rituximab. The administration of growth factors, i.e. G-CSF, should follow guidelines of the American Society of Oncology (ASCO). An antiinfective prophylaxis with trimethoprim/sulfmethoxazole was recommended (but not mandatory) from day 1 of study treatment until 2 months after the last day of the last treatment cycle. Antiviral prophylaxis was not recommended.

**Dose reduction rules**

In patients, whose blood counts had not recovered adequately within 28 days after start of a treatment cycle, the following cycle was allowed to be delayed for a maximum of 14 days. In case of a persisting cytopenia on day 42, a 50% dose reduction of fludarabine/cyclophosphamide was recommended for the given cycle. A discontinuation of study treatment was recommended if a severe infection (≥ grade 4) or other unacceptable toxicity occurred.

**Minimal residual disease assessment**

Minimal residual disease (MRD) was assessed per multicolor flow cytometry using carefuly validated assays, anticipating later published harmonization efforts from the European Research Initiative on CLL (ERIC).^[[2]](#footnote-2)^ Specifically, the 4-color MRD assay (**Table 1**) applied by the German CLL study group (central lab at the University Hospital in Kiel, responsible director: Dr. Sebastian Böttcher) has been validated with an ASO primer-based quanitative real-time IGH PCR in a total of 530 samples from the CLL8 trial, demonstrating its utility to detect MRD in CLL at a sensitivity of 10^-4^ as efficiently as molecular testing.^[[3]](#footnote-3)^

The MRD flow assay applied by the French Cooperative Group on CLL (central lab at the University Hospital Paris, responsible director: Dr. Rémi Letestu) used a6-color flow method (**Table 0**). Although the assay allowed detection of residual CLL cells at a sensitivity limit of less than 10^-4^, the results were also interpreted by applying a threshold of 10^-4^ (MRD negative: <0.01% CLL cells per leukocytes), in order to allow a combined evaluation of the German-French MRD datasets.

| **Tube** | **MRD flow panel GCLLSG** | **MRD flow panel FCGCLL** | **ERIC flow panel (1st International standardized approach, 2007)** |
| --- | --- | --- | --- |
| **# 1** | κ/λ/CD5/CD19 | κ/λ/CD19/CD5/CD79b/CD3+CD14 | κ/λ/CD5/CD19 |
| **# 2** | CD20/CD5/CD19/CD43 | CD43/CD22/CD19/CD5/CD81/CD3+CD14 | CD45/CD14/CD19/CD3 |
| **# 3** | CD81/CD22/CD19/CD5 | CD81/CD22/CD5/CD2+CD33/CD79b/CD19 | CD20/CD38/CD19/CD5 |
| **# 4** | CD79b/CD20/CD19/CD5 | n. a. | CD81/CD22/CD19/CD5 |
| **# 5** |  |  | CD43/CD79b/CD19/CD5 |

**Table 0:** Surface markers stained by the two MRD flow assays applied in the CLL7 study, in comparison to the first international standardized approach for MRD flow established by the European Research Initiative on CLL (ERIC) in 2007. The markers are sorted according to fluorescent dyes applied in the assay (not listed in this table for better readability). Underlined markers correspond to the mix of two antibodies detected in the same fluorescent channel used as a dump channel.

As illustrated in Table 0, most of the relevant CLL-associated markers (CD5, CD19, CD22, CD43, CD79b, CD81) were covered by both, the GCLLSG- and FCGCLL-panel, as well as the ERIC-recommended approach.^[[4]](#footnote-4)^ Comparable rates of patients achieving an MRD negative level at <10^-4^, e.g. in peripheral blood, were detected in the French and German patient cohorts (15/21 patients, 71.4% versus 25/32 patients, 78.1%, respectively).

**Responsibilities and data sharing**

This was an investigator initiated trial with legal sponsorship executed by the University of Cologne (Germany) and the French Cooperative Group on CLL (FCGCLL, France). The GCLLSG and FCGCLL were responsible for the study design, data collection, data cleaning, medical review, and data analysis. All authors were provided with access to the clinical data. Deidentified individual participant data that underlie the reported results will be made available for scientific non-commercial purposes upon request sent to the corresponding author, and after approval by the data owning study groups (GCLLSG/FCGCLL). The full study protocol can be accessed upon request to the GCLLSG study office (cll-studie@uk-koen.de).

**Supplemental Table 2: Second malignancies (all cohorts)**

| **Second malignancies** | | **N events** | | **N patients** | | | |
| --- | --- | --- | --- | --- | --- | --- | --- |
| **Overall** | | 29 | | 28 | | | |
| **Hi-FCR** | | 1 | | 1 | | | |
| **Hi-W&W** | | 4 | | 3 | | | |
| **Lo-W&W** | | 24 | | 24 | | | |
|  | | | | | | | |
| **Type of malignancy** | | **Hi-FCR** | **Hi-W&W** | | **Lo-W&W** | | **Total events** |
| **Hematologic malignancy** | | 1 | 0 | | 5 | | 6 |
| **Solid tumors (incl. melanoma)** | | 0 | 2 | | 16 | | 18 |
| **Basaliome/squamous cell tumor** | | 0 | 2 | | 3 | | 5 |
|  | | | | | | | |
| **Type of malignancy by study cohort/arm** | | | | | | | |
| **Study arm** | **Documented subtype of malignancy** | | **Classified type of malignancy^§^** | | | **Time of diagnosis (months from stratification/**  **randomization)** | |
| Hi - FCR | Acute myeloid leukemia | | Hematologic malignancy | | | 43.4 | |
| Hi - W&W | Lung cell cancer | | Solid tumor | | | 2.0 | |
| Hi - W&W | Adrenal gland tumor | | Solid tumor | | | 34.3 | |
| Hi - W&W | Spinalioma | | Basaliome, squamous cell | | | 58.9 | |
| Hi - W&W | Skin carcinoma | | Basaliome, squamous cell | | | 68.9 | |
| Lo - W&W | Basalioma | | Basaliome, squamous cell | | | 1.9 | |
| Lo - W&W | Breast cancer | | Solid tumor | | | 6.2 | |
| Lo - W&W | Gliosarcoma | | Solid tumor | | | 7.4 | |
| Lo - W&W | Adenocarcinoma, parotis gland | | Solid tumor | | | 8.9 | |
| Lo - W&W | Basal cell carcinoma | | Basaliome, squamous cell | | | 10.2 | |
| Lo - W&W | Acute myeloid leukemia | | Hematologic malignancy | | | 10.9 | |
| Lo - W&W | Prostated cancer | | Solid tumor | | | 27.1 | |
| Lo - W&W | Breast cancer | | Solid tumor | | | 27.3 | |
| Lo - W&W | Colon cancer | | Solid tumor | | | 27.9 | |
| Lo - W&W | Colon cancer | | Solid tumor | | | 29.3 | |
| Lo - W&W | Colon cancer | | Solid tumor | | | 30.8 | |
| Lo - W&W | Renal cancer | | Solid tumor | | | 36.7 | |
| Lo - W&W | Breast cancer | | Solid tumor | | | 38.7 | |
| Lo - W&W | Cutaneous precancerosis of skin | | Basaliome, squamous cell | | | 44.2 | |
| Lo - W&W | Pharyngal cancer | | Solid tumor | | | 45.9 | |
| Lo - W&W | Renal cancer | | Solid tumor | | | 46.6 | |
| Lo - W&W | Multiple myeloma | | Hematologic malignancy | | | 47.2 | |
| Lo - W&W | Breast cancer | | Solid tumor | | | 52.6 | |
| Lo - W&W | Melanoma | | Solid tumor | | | 57.5 | |
| Lo - W&W | Lung cancer | | Solid tumor | | | 57.6 | |
| Lo - W&W | Prostate cancer | | Solid tumor | | | 65.6 | |
| Lo - W&W | Acute myeloid leukemia | | Hematologic malignancy | | | 90.4 | |

^§^ Second malignancies (excluding Richter’s transformation) were classified as described previously by Maurer et al., Reference: Maurer C, Langerbeins P, Bahlo J, Cramer P, Fink AM, Pflug N, Engelke A, von Tresckow J, Kovacs G, Stilgenbauer S, Wendtner CM, Müller L, Ritgen M, Seiler T, Fischer K, Hallek M, Eichhorst B. Effect of first-line treatment on second primary malignancies and Richter's transformation in patients with CLL. Leukemia. 2016 Oct;30(10):2019-2025.

**Supplemental Figure 1: EFS in MRD-negative versus –positive patients as detected in bone marrow**

| **EFS from MRD landmark** | **Pts, N** | **Events, N** | **Median**  **months** | **3-year EFS, %** | **5-year EFS, %** |
| --- | --- | --- | --- | --- | --- |
| **MRD status in BM** | **28** | 5 |  |  |  |
| **Negative** | 19 | 2 | NR | 86.5 | 86.5 |
| **Positive** | 9 | 3 | NR | 66.7 | 66.7 |

*p* = 0.153 (non-stratified log-rank)

| **Number at risk** | **0** | **12** | **24** | **36** | **48** | **60** |
| --- | --- | --- | --- | --- | --- | --- |
| **Negative** | 19 | 18 | 18 | 12 | 5 | 2 |
| **Positive** | 9 | 8 | 7 | 5 | 1 | 1 |

| **COX regression EFS from landmark** | **Univariate comparison** | **Hazard ratio**  **[HR]** | **95% Confidence Interval** | | ***p* value** |
| --- | --- | --- | --- | --- | --- |
|  |  |  | **Lower** | **Upper** |  |
| **MRD status in BM** | | | | | |
| Positive | ***vs.*** negative | 3.409 | 0.569 | 20.427 | 0.179 |

Negative = < 10^-4^; positive = ≥ 10^-4^; NR, not reached

**Supplemental Table 3: Causes of death in patients classified as high-risk (Hi-FCR and Hi-W&W)**

| **Study arm** | **Number of early FCR cycles received** | **Overall survival from randomization (months)** | **Treatment status** | **Documented cause of death** | **Additional information provided by the investigator** |
| --- | --- | --- | --- | --- | --- |
| **Hi-FCR** | 4 | 9.26 | censored | Viral encephalitis | Prolonged neutropenia at the time of encephalitis, clinical radiologic diagnosis |
| **Hi-FCR** | 6 | 9.79 | censored | AIHA | n. a. |
| **Hi-FCR** | 0 | 11.47 | new treatment | Hemophagocytosis syndrome | Multimorbidity after allogeneic stem cell transplant with additional fungal pneumonia and chronic GvHD |
| **Hi-FCR** | 6 | 11.63 | censored | Sepsis | Preceding port catheter infection by multiresistant bacteria, no prolonged cytopenia |
| **Hi-FCR** | 2 | 12.16 | censored | Cerebral stroke | n. a. |
| **Hi-FCR** | 6 | 40.08 | new treatment | CLL | n. a. |
| **Hi-FCR** | 6 | 41.76 | censored | Pulmonary fibrosis | n. a. |
| **Hi-FCR** | 3 | 43.47 | censored | Aspiration pneumonia | Multimorbid patient with arterial hypertension, COPD, depression, atrial fibrillation, died in nursing home |
| **Hi-FCR** | 6 | 53.16 | censored | Richter transformation | Renal insufficiency |
| **Hi-FCR** | 6 | 57.99 | censored | Pneumopathy | n. a. |
| **Hi-FCR** | 6 | 58.71 | new treatment | Creutzfeld-Jakob-disease | Diagnosis was histologically confirmed by a cerebral biopsy, patient had been re-treated with bendamustine, rituximab |
| **Hi-FCR** | 1 | 61.86 | new treatment | Richter transformation | n. a. |
|  | | | | | |
| **Hi-W&W** | 0 | 4.50 | censored | Small cell lung cancer | n. a. |
| **Hi-W&W** | 0 | 6.14 | new treatment | CLL | n. a. |
| **Hi-W&W** | 0 | 9.10 | censored | Fulminant infection/pneumonia and sepsis | n. a. |
| **Hi-W&W** | 0 | 26.68 | new treatment | Respiratory insufficiency, atypical pneumonia | Chronic GvHD after allogeneic stem cell transplant |
| **Hi-W&W** | 0 | 31.87 | new treatment | Adverse event after new chemotherapy | Chronic GvHD after allogeneic stem cell transplant |
| **Hi-W&W** | 0 | 33.74 | censored | CLL | n. a. |
| **Hi-W&W** | 0 | 46.32 | new treatment | unknown | n. a. |
| **Hi-W&W** | 0 | 47.01 | new treatment | Secondary disease, not further specified (F2101) | n. a. |
| **Hi-W&W** | 0 | 51.35 | new treatment | CLL | n. a. |
| **Hi-W&W** | 0 | 53.32 | new treatment | Pneumonia during second line treatment | n. a. |
| **Hi-W&W** | 0 | 54.97 | new treatment | Infection (F609) | n. a. |

**Supplemental Figure 2:** **OS in MRD-negative versus –positive patients as detected in bone marrow**

| **OS from landmark** | **Pts, N** | **Events, N** | **Median**  **months** | **3-year OS, %** | **5-year OS, %** |
| --- | --- | --- | --- | --- | --- |
| **MRD status in BM** | **28** | 1 |  |  |  |
| **Negative** | 19 | 1 | NR | 94.1 | 94.1 |
| **Positive** | 9 | 0 | NR | 100.0 | 100.0 |

NR, not reached

*p* = 0.493 (log-rank)

| **Number at risk** | **0** | **12** | **24** | **36** | **48** | **60** |
| --- | --- | --- | --- | --- | --- | --- |
| **Negative** | 19 | 18 | 18 | 14 | 5 | 2 |
| **Positive** | 9 | 9 | 9 | 7 | 3 | 1 |

***p* = 0.493 (log-rank)**

The cox regression model is not applicable since all cases are censored for patients with positive MRD status.

**Supplemental Figure 3: PFS (comparison of all study cohorts based on ITT)**

| **PFS** | **Pts,**  **N** | **Events,**  **N (%)** | **Median**  **months** | **2-year Survival, %** | **3-year Survival, %** | **5-year Survival, %** |
| --- | --- | --- | --- | --- | --- | --- |
| **All patients [ITT]** | **800** | 244 |  |  |  |  |
| **Lo-W&W** | 599 | 128 (21.4) | 84.3 | 88.8 | 84.3 | 77.1 |
| **Hi-FCR** | 100 | 36 (36.0) | NR | 85.3 | 77.4 | 55.2 |
| **Hi-W&W** | 101 | 80 (79.2) | 18.6 | 39.7 | 23.3 | 12.6 |

NR, not reached

*p* < 0.001 (non-stratified log-rank)

| **Number at risk** | **0** | **12** | **24** | **36** | **48** | **60** | **72** | **84** |
| --- | --- | --- | --- | --- | --- | --- | --- | --- |
| **Lo-W&W** | 599 | 524 | 474 | 418 | 323 | 180 | 36 | 5 |
| **Hi-FCR** | 100 | 86 | 79 | 63 | 40 | 18 | 4 | 1 |
| **Hi-W&W** | 101 | 61 | 37 | 18 | 9 | 4 | 2 | - |

| **COX regression PFS** | **Univariate comparison** | **Hazard ratio**  **[HR]** | **95% Confidence Interval** | | ***p* value** |
| --- | --- | --- | --- | --- | --- |
|  |  |  | **Lower** | **Upper** |  |
| **Study cohort** | | | | | |
| Hi-FCR | ***vs.*** LR | 1.876 | 1.295 | 2.718 | 0.001 |
| Hi-W&W | ***vs.*** LR | 8.131 | 6.088 | 10.860 | < 0.001 |

**Supplemental Figure 4:** **EFS for patients with trisomy12 (high-risk cohorts)**

| **EFS** | **Pts,**  **N (%)** | **Events, N** | **Median**  **months** | **3-year EFS, %** | **5-year EFS, %** |
| --- | --- | --- | --- | --- | --- |
| **Patients with trisomy 12**^1^ | **49** |  |  |  |  |
| **Hi-FCR** | 25 | 6 | NR | 79.2 | 74.2 |
| **Hi-W&W** | 24 | 17 | 19.4 | 34.1 | 22.7 |

NR, not reached; ^1^ according to Döhner et al. NEJM 2000

*p* < 0.001 (log-rank)

| **Number at risk** | **0** | **12** | **24** | **36** | **48** | **60** | **72** | **84** |
| --- | --- | --- | --- | --- | --- | --- | --- | --- |
| **Hi-FCR** | 25 | 22 | 20 | 18 | 12 | 7 | 2 | 1 |
| **Hi-W&W** | 24 | 16 | 11 | 6 | 3 | 1 | 1 | 0 |

| **COX regression EFS** | **Univariate comparison** | **Hazard ratio**  **[HR]** | **95% Confidence Interval** | | ***p* value** |
| --- | --- | --- | --- | --- | --- |
|  |  |  | **Lower** | **Upper** |  |
| **Study cohort** | | | | | |
| Hi-FCR | ***vs.*** Hi-W&W | 0.2 | 0.08 | 0.5 | 0.001 |

**Supplemental Figure 5:** **OS for *IGHV* mutated patients (high-risk cohorts)**

| **OS** | **Pts,**  **N (%)** | **Events, N** | **Median**  **months** | **3-year EFS, %** | **5-year EFS, %** |
| --- | --- | --- | --- | --- | --- |
| **Mutated *IGHV*** | **37** |  |  |  |  |
| **Hi-FCR** | 19 | 1 | NR | 100.0 | 91.7 |
| **Hi-W&W** | 18 | 1 | NR | 92.3 | 92.3 |

NR, not reached

*p* = 0.873 (log-rank)

| **Number at risk** | **0** | **12** | **24** | **36** | **48** | **60** | **72** | **84** |
| --- | --- | --- | --- | --- | --- | --- | --- | --- |
| **Hi-FCR** | 19 | 18 | 17 | 15 | 11 | 4 | 0 | - |
| **Hi-W&W** | 18 | 17 | 16 | 12 | 7 | 4 | 3 | 0 |

| **COX regression OS** | **Univariate comparison** | **Hazard ratio**  **[HR]** | **95% Confidence Interval** | | ***p* value** |
| --- | --- | --- | --- | --- | --- |
|  |  |  | **Lower** | **Upper** |  |
| **Study cohort** | | | | | |
| Hi-FCR | ***vs.*** Hi-W&W | 0.8 | 0.05 | 12.8 | 0.873 |

**Supplemental Table 4:** **Distribution of CLL-IPI risk in the study cohorts**

|  | **Hi-Risk**  **FCR**  **N (%)** | **High-Risk W&W**  **N (%)** | **Low Risk**  **N (%)** | **Total**  **N (%)** |
| --- | --- | --- | --- | --- |
| **CLL-IPI risk group, N (%)*** | 40 | 39 | 169 | 248 |
| Low | 11 (27.5) | 8 (20.5) | 150 (88.8) | 169 (68.1) |
| Intermediate | 27 (67.5) | 24 (61.5) | 18 (10.7) | 69 (27.8) |
| High | 1 (2.5) | 7 (17.9) | 1 (0.6) | 9 (3.6) |
| Very high | 1 (2.5) | 0 (0.0) | 0 (0.0) | 1 (0.4) |
|  |  |  |  |  |
| Missing information | 60 (60.0) | 62 (61.4) | 430 (71.8) | 552 (69.0) |

*****Beta-2-microglobuline (β2M) was not assessed centrally in the French patient cohort. Also screening for *TP53* mutations was not established as a standard assessment at the time the trial was initiated. If we calculate the CLL-IPI using only del(17p) as a marker for *TP53* aberrations, we overall yield 69% of patients with missing information, mostly due to unavailable β2M. Therefore, the comparison of our risk stratification with the CLL-IPI is only limited informative. However, from the available data we can at least deduct, that our risk stratification approximated a separation between CLL-IPI low risk versus CLL-IPI intermediate/high/very high risk. About 89% of low risk patients per our study were also categorized low-risk per CLL-IPI. Vice versa about two third of our high-risk cohorts were at least intermediate risk per CLL-IPI.

**Supplemental Table 5:** **First-line therapies administered in Hi-W&W**

| **Patient ID** | **Study cohort/arm** | **Type of first-line therapy (as documented)** |
| --- | --- | --- |
| 103 | HR-W&W | FCR |
| 111 | HR-W&W | FCR |
| 112 | HR-W&W | FCR |
| 126 | HR-W&W | FCR |
| 138 | HR-W&W | FCR |
| 307 | HR-W&W | FCR |
| 513 | HR-W&W | FCR |
| 527 | HR-W&W | FCR |
| 530 | HR-W&W | ALEMTUZUMAB |
| 609 | HR-W&W | ALEMTUZUMAB / DEXAMETHASONE, ALLOGRAFT |
| 612 | HR-W&W | FCR |
| 618 | HR-W&W | ALEMTUZUMAB, ALLOGRAFT |
| 622 | HR-W&W | FCR |
| 901 | HR-W&W | FCR |
| 910 | HR-W&W | FCR |
| 918 | HR-W&W | ALEMTUZUMAB |
| 923 | HR-W&W | FCR |
| 1401 | HR-W&W | FCR |
| 1501 | HR-W&W | FCR |
| 1704 | HR-W&W | FCR |
| 1815 | HR-W&W | FCR |
| 1902 | HR-W&W | FCR |
| 1910 | HR-W&W | FCR |
| 1921 | HR-W&W | FCR |
| 1925 | HR-W&W | FCR, RITUXIMAB MAINTENANCE |
| 2008 | HR-W&W | ALEMTUZUMAB / DEXAMETHASONE |
| 2014 | HR-W&W | BR |
| 2101 | HR-W&W | CHEMOTHERAPY (not further specified) |
| 2414 | HR-W&W | FCR |
| 9004 | HR-W&W | FLUDARABINE / CYCLOPHOSPHAMIDE / OFATUMUMAB |
| 9007 | HR-W&W | FLUDARABINE / CYCLOPHOSPHAMIDE / OFATUMUMAB |
| 9017 | HR-W&W | FCR |
| 9018 | HR-W&W | BR |
| 15015 | HR-W&W | FCR |
| 15017 | HR-W&W | FCR |
| 18001 | HR-W&W | RITUXIMAB / CYCLOPHOSPHAMIDE |
| 41023 | HR-W&W | FCR |
| 145001 | HR-W&W | FCR |
| 170003 | HR-W&W | CHLORAMBUCIL |
| 210002 | HR-W&W | CHLORAMBUCIL |
| 258004 | HR-W&W | FCR |
| 258010 | HR-W&W | FCR |
| 280001 | HR-W&W | FCR |
| 305002 | HR-W&W | FCR |
| 305012 | HR-W&W | BR |
| 313002 | HR-W&W | BENDAMUSTINE |
| 313003 | HR-W&W | BR |
| 334001 | HR-W&W | R-CHOP |
| 334023 | HR-W&W | BR |
| 334028 | HR-W&W | BR |
| 334033 | HR-W&W | BR |
| 415005 | HR-W&W | FCR |
| 415009 | HR-W&W | FCR |
| 421004 | HR-W&W | FCR |
| 425001 | HR-W&W | FCR |
| 438001 | HR-W&W | ALEMTUZUMAB |
| 454001 | HR-W&W | BR |
| 461004 | HR-W&W | FCR |
| 461008 | HR-W&W | FCR |
| 483001 | HR-W&W | FCR |
| 500001 | HR-W&W | FCR |
| 503001 | HR-W&W | VINBLASTIN |
| 701001 | HR-W&W | RITUXIMAB / CYCLOPHOSPHAMIDE / DEXAMETHASONE |
| 701017 | HR-W&W | FCR |
| 781001 | HR-W&W | RITUXIMAB / CHLORAMBUCIL |
| 781005 | HR-W&W | GA101 / CHLORAMBUCIL |
| 1182001 | HR-W&W | FCR |
| 1182004 | HR-W&W | FCR |
| 1182005 | HR-W&W | BR |
| 1187002 | HR-W&W | FCR |

**Abbreviations:** BR = combined bendamustine / rituximab; FCR = combined fludarabine / cyclophosphamide / rituximab, other treatments are further specified

**Supplemental Figure 6:** **EFS for *IGHV* mutated patients (high-risk cohorts)**

| **EFS** | **Pts,**  **N (%)** | **Events, N** | **Median**  **months** | **3-year EFS, %** | **5-year EFS, %** |
| --- | --- | --- | --- | --- | --- |
| **Mutated IGHV** | **37** |  |  |  |  |
| **Hi-FCR** | 19 | 4 | NR | 89.2 | 73.0 |
| **Hi-W&W** | 18 | 13 | 17.9 | 28.2 | 21.2 |

NR, not reached

*p* < 0.001 (log-rank)

| **Number at risk** | **0** | **12** | **24** | **36** | **48** | **60** | **72** | **84** |
| --- | --- | --- | --- | --- | --- | --- | --- | --- |
| **Hi-FCR** | 19 | 17 | 15 | 14 | 9 | 4 | 0 | - |
| **Hi-W&W** | 18 | 11 | 8 | 4 | 2 | 1 | 1 | - |

| **COX regression EFS** | **Univariate comparison** | **Hazard ratio**  **[HR]** | **95% Confidence Interval** | | ***p* value** |
| --- | --- | --- | --- | --- | --- |
|  |  |  | **Lower** | **Upper** |  |
| **Study cohort** | | | | | |
| Hi-FCR | ***vs.*** Hi-W&W | 0.2 | 0.05 | 0.5 | 0.001 |

**Supplemental Figure 7:** **EFS for *IGHV* unmutated patients (high-risk cohorts)**

| **EFS** | **Pts,**  **N (%)** | **Events, N** | **Median**  **months** | **3-year EFS, %** | **5-year EFS, %** |
| --- | --- | --- | --- | --- | --- |
| **Unmutated IGHV** | **163** |  |  |  |  |
| **Hi-FCR** | 81 | 32 | 60.3 | 74.7 | 50.6 |
| **Hi-W&W** | 82 | 69 | 17.3 | 21.9 | 10.9 |

*p* < 0.001 (log-rank)

| **Number at risk** | **0** | **12** | **24** | **36** | **48** | **60** | **72** | **84** |
| --- | --- | --- | --- | --- | --- | --- | --- | --- |
| **Hi-FCR** | 81 | 69 | 64 | 49 | 31 | 14 | 4 | 1 |
| **Hi-W&W** | 82 | 49 | 28 | 14 | 7 | 4 | 1 | 0 |

| **COX regression EFS** | **Univariate comparison** | **Hazard ratio**  **[HR]** | **95% Confidence Interval** | | ***p* value** |
| --- | --- | --- | --- | --- | --- |
|  |  |  | **Lower** | **Upper** |  |
| **Study cohort** | | | | | |
| Hi-FCR | ***vs.*** Hi-W&W | 0.2 | 0.15 | 0.4 | < 0.001 |

**Supplemental Figure 8:** **EFS for patients with del(11q) (high-risk cohorts)**

| **EFS** | **Pts,**  **N (%)** | **Events, N** | **Median**  **months** | **3-year EFS, %** | **5-year EFS, %** |
| --- | --- | --- | --- | --- | --- |
| **Patients with del(11q)^[[5]](#footnote-5)^** | **52** |  |  |  |  |
| **Hi-FCR** | 17 | 6 | 55.6 | 81.9 | 46.1 |
| **Hi-W&W** | 35 | 29 | 15.8 | 22.8 | 13.7 |

NR, not reached; ^1^ according to Döhner et al. NEJM 2000

*p* < 0.001 (log-rank)

| **Number at risk** | **0** | **12** | **24** | **36** | **48** | **60** | **72** | **84** |
| --- | --- | --- | --- | --- | --- | --- | --- | --- |
| **Hi-FCR** | 17 | 16 | 15 | 10 | 7 | 3 | 1 | 0 |
| **Hi-W&W** | 35 | 22 | 14 | 6 | 2 | 2 | 0 | - |

| **COX regression EFS** | **Univariate comparison** | **Hazard ratio**  **[HR]** | **95% Confidence Interval** | | ***p* value** |
| --- | --- | --- | --- | --- | --- |
|  |  |  | **Lower** | **Upper** |  |
| **Study cohort** | | | | | |
| Hi-FCR | ***vs.*** Hi-W&W | 0.2 | 0.09 | 0.5 | 0.001 |

**Supplemental Figure 9:** **EFS for patients with del(17p) (high-risk cohorts)**

| **EFS** | **Pts,**  **N (%)** | **Events, N** | **Median**  **months** | **3-year EFS, %** | **5-year EFS, %** |
| --- | --- | --- | --- | --- | --- |
| **Patients with del(17p)^1^** | **13** |  |  |  |  |
| **Hi-FCR** | 4 | 3 | 9.8 | 25.0 | 25.0^2^ |
| **Hi-W&W** | 9 | 9 | 20.8 | 11.1 | 0.0^2^ |

NR, not reached; ^1^ according to Döhner et al. NEJM 2000; ^2^ at last observation (month 36.2 and 56.9, respectively)

*p* = 0.771 (log-rank)

| **Number at risk** | **0** | **12** | **24** | **36** | **48** | **60** | **72** | **84** |
| --- | --- | --- | --- | --- | --- | --- | --- | --- |
| **Hi-FCR** | 4 | 2 | 1 | 1 | 1 | 0 | - | - |
| **Hi-W&W** | 9 | 5 | 2 | 1 | 0 | - | - | - |

| **COX regression EFS** | **Univariate comparison** | **Hazard ratio**  **[HR]** | **95% Confidence Interval** | | ***p* value** |
| --- | --- | --- | --- | --- | --- |
|  |  |  | **Lower** | **Upper** |  |
| **Study cohort** | | | | | |
| Hi-FCR | ***vs.*** Hi-W&W | 0.8 | 0.2 | 3.1 | 0.771 |

**Supplemental Figure 10**: **EFS for patients without del(11q) / del(17p) / trisomy 12 (high-risk cohorts)**

| **EFS** | **Pts,**  **N (%)** | **Events, N** | **Median**  **months** | **3-year EFS, %** | **5-year EFS, %** |
| --- | --- | --- | --- | --- | --- |
| **Patients without**  **del(11q) / del(17p) / trisomy 12** | **86** |  |  |  |  |
| **Hi-FCR** | 54 | 21 | 60.3 | 79.1 | 50.6 |
| **Hi-W&W** | 32 | 27 | 13.3 | 17.5 | 10.5 |

NR, not reached

*p* < 0.001 (log-rank)

| **Number at risk** | **0** | **12** | **24** | **36** | **48** | **60** | **72** | **84** |
| --- | --- | --- | --- | --- | --- | --- | --- | --- |
| **Hi-FCR** | 54 | 46 | 43 | 34 | 20 | 8 | 1 | 0 |
| **Hi-W&W** | 32 | 17 | 9 | 5 | 4 | 2 | 1 | 0 |

| **COX regression EFS** | **Univariate comparison** | **Hazard ratio**  **[HR]** | **95% Confidence Interval** | | ***p* value** |
| --- | --- | --- | --- | --- | --- |
|  |  |  | **Lower** | **Upper** |  |
| **Study cohort** | | | | | |
| Hi-FCR | ***vs.*** Hi-W&W | 0.2 | 0.1 | 0.4 | < 0.001 |

**Supplemental Figure 11:** **OS for *IGHV* unmutated patients (high-risk cohorts)**

| **OS** | **Pts,**  **N (%)** | **Events, N** | **Median**  **months** | **3-year EFS, %** | **5-year EFS, %** |
| --- | --- | --- | --- | --- | --- |
| **Unmutated *IGHV*** | **163** |  |  |  |  |
| **Hi-FCR** | 81 | 11 | NR | 93.4 | 80.7 |
| **Hi-W&W** | 82 | 10 | NR | 93.4 | 77.1 |

NR, not reached

*p* = 0.878 (log-rank)

| **Number at risk** | **0** | **12** | **24** | **36** | **48** | **60** | **72** | **84** |
| --- | --- | --- | --- | --- | --- | --- | --- | --- |
| **Hi-FCR** | 81 | 72 | 71 | 63 | 45 | 22 | 5 | 2 |
| **Hi-W&W** | 82 | 76 | 71 | 56 | 31 | 15 | 7 | 0 |

| **COX regression OS** | **Univariate comparison** | **Hazard ratio**  **[HR]** | **95% Confidence Interval** | | ***p* value** |
| --- | --- | --- | --- | --- | --- |
|  |  |  | **Lower** | **Upper** |  |
| **Study cohort** | | | | | |
| Hi-FCR | ***vs.*** Hi-W&W | 0.9 | 0.4 | 2.2 | 0.878 |

**Supplemental Figure 12:** **OS for patients with del(11q) (high-risk cohorts)**

| **OS** | **Pts,**  **N (%)** | **Events, N** | **Median**  **months** | **3-year EFS, %** | **5-year EFS, %** |
| --- | --- | --- | --- | --- | --- |
| **Patients with del(11q)**^1^ | **52** |  |  |  |  |
| **Hi-FCR** | 17 | 1 | NR | 94.1 | 94.1 |
| **Hi-W&W** | 35 | 4 | NR | 90.5 | 79.2 |

NR, not reached; ^1^ according to Döhner et al. NEJM 2000

*p* = 0.473 (log-rank)

| **Number at risk** | **0** | **12** | **24** | **36** | **48** | **60** | **72** | **84** |
| --- | --- | --- | --- | --- | --- | --- | --- | --- |
| **Hi-FCR** | 17 | 16 | 16 | 12 | 8 | 3 | 1 | 0 |
| **Hi-W&W** | 35 | 32 | 31 | 22 | 10 | 4 | 1 | 0 |

| **COX regression EFS** | **Univariate comparison** | **Hazard ratio**  **[HR]** | **95% Confidence Interval** | | ***p* value** |
| --- | --- | --- | --- | --- | --- |
|  |  |  | **Lower** | **Upper** |  |
| **Study cohort** | | | | | |
| Hi-FCR | ***vs.*** Hi-W&W | 0.2 | 0.05 | 4.1 | 0.484 |

**Supplemental Figure 13:** **OS for patients with del(17p) (high-risk cohorts)**

| **OS** | **Pts,**  **N (%)** | **Events, N** | **Median**  **months** | **3-year EFS, %** | **5-year EFS, %** |
| --- | --- | --- | --- | --- | --- |
| **Patients with del(17p)**^1^ | **13** |  |  |  |  |
| **Hi-FCR** | 4 | 3 | 11.5 | 25.0 | 25.0^2^ |
| **Hi-W&W** | 9 | 4 | 55.0 | 77.8 | 31.1 |

^1^ according to Döhner et al. NEJM 2000

*p* = 0.139 (log-rank)

| **Number at risk** | **0** | **12** | **24** | **36** | **48** | **60** | **72** | **84** |
| --- | --- | --- | --- | --- | --- | --- | --- | --- |
| **Hi-FCR** | 4 | 2 | 1 | 1 | 1 | 0 | - | - |
| **Hi-W&W** | 9 | 9 | 9 | 7 | 4 | 1 | 1 | 0 |

| **COX regression EFS** | **Univariate comparison** | **Hazard ratio**  **[HR]** | **95% Confidence Interval** | | ***p* value** |
| --- | --- | --- | --- | --- | --- |
|  |  |  | **Lower** | **Upper** |  |
| **Study cohort** | | | | | |
| Hi-FCR | ***vs.*** Hi-W&W | 3.1 | 0.6 | 14.4 | 0.157 |

**Supplemental Figure 14:** **OS for patients with trisomy 12 (high-risk cohorts)**

| **OS** | **Pts,**  **N (%)** | **Events, N** | **Median**  **months** | **3-year EFS, %** | **5-year EFS, %** |
| --- | --- | --- | --- | --- | --- |
| **Patients with trisomy 12**^1^ | **49** |  |  |  |  |
| **Hi-FCR** | 25 | 1 | NR | 100.0 | 95.0 |
| **Hi-W&W** | 24 | 0 | NR | 100.0 | 100.0 |

NR, not reached; ^1^ according to Döhner et al. NEJM 2000;

*p* = 0.386 (log-rank)

| **Number at risk** | **0** | **12** | **24** | **36** | **48** | **60** | **72** | **84** |
| --- | --- | --- | --- | --- | --- | --- | --- | --- |
| **Hi-FCR** | 25 | 24 | 24 | 22 | 14 | 8 | 2 | 0 |
| **Hi-W&W** | 24 | 23 | 20 | 16 | 11 | 5 | 3 | 0 |

| **COX regression OS** | **Univariate comparison** | **Hazard ratio**  **[HR]** | **95% Confidence Interval** | | ***p* value** |
| --- | --- | --- | --- | --- | --- |
|  |  |  | **Lower** | **Upper** |  |
| **Study cohort** | | | | | |
| Hi-FCR | ***vs.*** Hi-W&W | 50.5 | 0.00 | *NE* | 0.638 |

*NE*, not evaluable

**Supplemental Figure 15:** **OS for patients without del(11q) / del(17p) / trisomy 12 (high-risk cohorts)**

| **OS** | **Pts,**  **N (%)** | **Events, N** | **Median**  **months** | **3-year EFS, %** | **5-year EFS, %** |
| --- | --- | --- | --- | --- | --- |
| **Patients without del(11q)/del(17p)/trisomy 12** | **86** |  |  |  |  |
| **Hi-FCR** | 54 | 7 | NR | 98.0 | 81.0 |
| **Hi-W&W** | 32 | 3 | NR | 96.8 | 83.2 |

NR, not reached

*p* = 0.757 (log-rank)

| **Number at risk** | **0** | **12** | **24** | **36** | **48** | **60** | **72** | **84** |
| --- | --- | --- | --- | --- | --- | --- | --- | --- |
| **HR-FCR** | 54 | 48 | 47 | 43 | 33 | 15 | 2 | 1 |
| **HR-W&W** | 32 | 29 | 27 | 23 | 13 | 9 | 5 | 0 |

| **COX regression OS** | **Univariate comparison** | **Hazard ratio**  **[HR]** | **95% Confidence Interval** | | ***p* value** |
| --- | --- | --- | --- | --- | --- |
|  |  |  | **Lower** | **Upper** |  |
| **Study cohort** | | | | | |
| Hi-FCR | ***vs.*** Hi-W&W | 1.2 | 0.3 | 4.8 | 0.758 |

1. Hoechstetter MA, Busch R, Eichhorst B, et al. Early, risk-adapted treatment with fludarabine in Binet stage A chronic lymphocytic leukemia patients: results of the CLL1 trial of the German CLL study group. Leukemia. 2017;31(12):2833-2837. [↑](#footnote-ref-1)
2. Rawstron AC, Villamor N, Ritgen M, Bottcher S, Ghia P, Zehnder JL, et al. International standardized approach for flow cytometric residual disease monitoring in chronic lymphocytic leukaemia. Leukemia. 2007;21(5):956-64. [↑](#footnote-ref-2)
3. Bottcher S, Stilgenbauer S, Busch R, Bruggemann M, Raff T, Pott C, et al. Standardized MRD flow and ASO IGH RQ-PCR for MRD quantification in CLL patients after rituximab-containing immunochemotherapy: a comparative analysis. Leukemia. 2009;23(11):2007-17. [↑](#footnote-ref-3)
4. Rawstron AC, Villamor N, Ritgen M, Bottcher S, Ghia P, Zehnder JL, et al. International standardized approach for flow cytometric residual disease monitoring in chronic lymphocytic leukaemia. Leukemia. 2007;21(5):956-64. [↑](#footnote-ref-4)
5. [↑](#footnote-ref-5)
